# Effects of topical insulin on second-intention wound healing in the red-eared slider turtle (*Trachemys scripta elegans*) – a controlled study

**DOI:** 10.1186/s12917-017-1082-8

**Published:** 2017-06-06

**Authors:** Joao Negrini, Elena Mozos, Alejandro Escamilla, José Pérez, Rosario Lucena, Rafael Guerra, Pedro J. Ginel

**Affiliations:** 1 Faculty of Veterinary, Medicine University of Córdoba, Campus de Rabanales, 14014 Córdoba, Spain; 2 Department of Animal Medicine & Surgery, Faculty of Veterinary Medicine, University of Córdoba, Campus de Rabanales, 14014 Córdoba, Spain; 3 Department of Veterinary Medicine, Federal University of Mato Grosso do Sul, Cidade Universitaria, Campo Grande, 79070-900 Brazil; 4 Zoological Garden of Córdoba, Avenida de Linneo, 14071 Córdoba, Spain

**Keywords:** *Trachemys scripta*, Wound healing, Topical insulin, Skin, Reptile

## Abstract

**Background:**

Compared with mammals, wound healing in reptiles is characterized by reduced wound contraction and longer healing times. The aim of this study is to describe the clinical and histopathological effects of topical insulin on second-intention healing of experimentally induced wounds in skin without dermal bony plates of *Trachemys scripta elegans* exposed to daily variations in ambient temperature and in an aquatic environment. Forty-four healthy adult females were assigned to two groups: Group 1 (*n* = 24) was used to assess clinical features such as wound contraction; Group 2 (*n* = 20) was used for histological evaluation and morphometric analysis. Topical porcine insulin (5 IU/ml diluted in glycerol) was applied daily 1 week. For each control time (2, 7, 14, 21 and 28 days post-wounding), re-epithelisation and wound remodelling were evaluated histologically and the number of main inflammatory cells (heterophils, macrophages, lymphocytes and fibroblasts) was scored.

**Results:**

Mean wound contraction was higher in the insulin-treated group at each time point and differences were significant at day 28 (*P* < 0.0001). Histologically, these clinical findings were associated with better re-epithelisation, inflammatory response, collagen synthesis and remodelling of the wounds. Morphometrically, insulin-treated wounds had significantly higher mean counts of heterophils (day 7), macrophages (days 2, 7 and 14) and fibroblasts (days 14 and 21), whereas lymphocyte counts were significantly lower at day 21. These results demonstrate that topical insulin modifies the inflammatory response of turtle skin up-regulating inflammatory cells at early stages and promoting wound healing.

**Conclusions:**

Topical insulin is a potentially useful therapy in skin wounds of *Trachemys scripta* and should be evaluated in non-experimental wounds of turtles and other reptiles.

## Background

Skin wounds are a common reason for veterinary consultation in reptiles kept in captivity. The list of potential causes includes thermal burns, trauma, surgery, improper husbandry, unsanitary conditions, and stress among others [1–4]. Compared with mammals, skin wounds of reptiles heal more slowly and their rate of healing is influenced by ambient temperature [5, 6]. These features predispose reptiles to secondary infections, chronic ulcers and extended treatment times. Chronic or severe skin wounds, resulting in damage of both parenchyma cell and stromal framework of the tissue, require second-intention wound healing which is defined as a healing process by deposition of collagen and other extra cellular matrix causing formation of a scar [7–9]. In reptiles, skin healing is characterized by reduced wound contraction [3, 6, 10], which further compromises wound healing in these species. In consequence, there is a need for practical treatments to increase skin wound contraction and improve overall wound healing in reptiles.

Insulin is a peptide hormone and growth factor with several important physiological roles. In addition to its primary function in maintaining glucose blood levels and protein synthesis, insulin also participates in cell differentiation, with important metabolic and mitogenic effects mediated by an insulin receptor present in virtually all vertebrate tissues [11]. In reptiles, several studies have confirmed the presence of insulin producing cells in the pancreas and intestine of different species including chelonians [12–14]. The chemical nature of chelonian insulin has not been determined but the same antiserum against porcine insulin cross-reacted with β cells in *Chrysemys* (now named *Trachemys*) and in other chelonians’ pancreas as well as in mouse and rat pancreas [13].

Several studies have evaluated the clinical applications of growth factors, especially in the field of wound healing. Unfortunately, the high cost of producing purified growth factors has prevented their integration into standard therapies in veterinary practice. Recently, a convenient protocol to concentrate platelets from chelonian whole blood has been described and the thrombocyte enriched plasma obtained was used successfully to treat several clinical cases of traumatic injuries [4]. As another alternative, insulin has been reported to promote wound healing in mammals and, in contrast to other growth factors, is affordable and readily available worldwide [15, 16]. Some experimental models have shown that topical insulin application promoted healing of thermal traumas and incision wounds in rats and mice [11, 17, 18].

A review of the literature demonstrated that, despite evidence of a significant role for topical insulin in the promotion of wound healing in several animal models, few works have been focused in reptiles. The red-eared slider is a terrapin widely distributed in North America as a wild reptile. It is also common in Europe as an imported pet what makes this species suitable as a model for the study of wound healing in chelonians and other reptiles. The aim of this study was to describe the clinical and histopathological effects of topical porcine insulin in the second-intention wound healing of experimentally induced wounds in skin without bony plates in the dermis of *Trachemys scripta elegans* exposed to daily variations in ambient temperature and in an aquatic environment.

## Methods

### Animals

Forty-four *Trachemys scripta elegans* adult females (range of weight 1.2 to 2.3 kg; straight carapace length ≥ 16 cm) [19] were used. All animals were captive bred and belonged to a zoological garden collection. Prior to entering the study a physical examination, packed cell volume (normal range 25 to 33%) [20] and faecal flotation analysis were carried out. Turtles considered healthy were individually identified with a microchip and housed outdoor in nine *vivariums* with an area of 3 m^2^; every *vivarium* included a plastic pool with a capacity of 900 L that allowed for complete submersion. Water was changed daily and was obtained from the public water service that carried out physicochemical and microbiological controls twice monthly. They had free access to a sunbathing area and were fed *ad libitum* with a commercial diet (Aquatic Turtle Monster Diet, Zeigler Bros, Inc., Gardners, PA 17324, USA).

After a period for adaptation, the animals were distributed in two groups: Group 1, with 24 animals, was used to assess clinical features such as wound contraction; Group 2, with 20 animals, was used for histological evaluation in which sets of wounds were biopsied at defined time points along the cicatrization process. Both groups shared the same premises and climate conditions (mean ± SD nocturnal and diurnal temperatures 16.3 ± 1.9 °C and 32.7 ± 3.1 °C respectively; humidity 45–58%), that were considered adequate to study wound healing because corresponded with the appropriate temperature range (ATR) for these freshwater turtles [21].

### Skin wound biopsy

The animals were anaesthetized with ketamine (20 mg/kg intramuscularly (IM); Imalgene® 100 mg/ml, Merial, Barcelona, Spain) and medetomidine (0.5 mg/kg IM; Domtor® 1 mg/ml, Lab. Esteve, Barcelona, Spain) both injected on the front legs. Without previous disinfection, one wound was made on the dorsal aspect of each rear limb using a disposable circular scalpel 6 mm in diameter. After removing the biopsy piece the wounds had well-formed circular walls and reached the subcutaneous skeletal muscles and blood vessels. The wounds were made symmetrically because previous studies in rats have shown differential healing of cutaneous wounds in the same animal depending on their cranial-caudal location [22].

After every procedure the animals were housed in individual *terrariums* at room temperature for approximately 12 h. Following this recovery period, the animals did not show signs of discomfort and were returned to their *vivarium*. Local anaesthesia and post biopsy analgesia or anti-inflammatory therapies were not administered to avoid their impact on wound healing. Haemorrhage was minimal in all turtles and controlled with digital pressure. The skin biopsy samples obtained at wound-induction time were fixed in 10% formaldehyde during 16 to 20 h, processed to paraffin-embedded, stained according to routine histological procedures and used as control of normal skin.

### Insulin treatment

Porcine insulin (Caninsulin® 40 IU/mL, MSD Animal Health, Salamanca, Spain) at 5 IU/ml diluted in glycerol (G5516, Sigma-Aldrich, Inc., Missouri, USA) was administered topically 6 h after wound induction and daily during the first week post-injury. Allocation of treatments alternated between right and left rear limbs and the control wound received only topical glycerol. After each application, the animals were kept out of the water for a period of 1 h.

### Clinical evaluation of wound healing

Clinical evaluation of wound contraction and overall healing process was performed in 48 wounds from Group 1 (24 insulin-treated and 24 controls). Wounds were photographed on day 0 and weekly until 28 days post-wound (DPW) (time points T0 to T4, respectively) using a macro lens (Nikon AF-S DX 40 mm). After this four-week period, image analysis software (Analysing digital imaging; Global System Science; University of California, USA) was used to measure wound perimeter at each time point. Wound contraction was expressed as the percentage of perimeter reduction from the initial wound. Other measurements, such as area and diameter, were evaluated and provided comparable information. The photographs were examined by two observers, blind for animal and time point of each wound. The correlation coefficient between observers was 0.98 as calculated from 20 repeated measures of wound perimeter.

### Histological study

Forty wounds (20 insulin-treated and 20 control; Group 2) were used for the microscopic study; 8 wounds, 4 treated and 4 control, selected using a computational random number generator, were re-biopsied under general anaesthesia at 2, 7, 14, 21 and 28 DPW, using a circular scalpel 8 mm in diameter. After this procedure, wounds were closured and the turtles kept in the *vivariums* under observation until sutures were removed and the animals returned to their pond. Samples were fixed in 10% formaldehyde during 16 to 20 h, then, cut across into two halves and processed to paraffin-embedded. Four to 5 μm thick serial sections were obtained from each block. Sections were stained with hematoxylin and eosin (HE) to evaluate the microscopic features, Fraser-Lendrum (FL) to identify fibrin exudate, methenamine silver staining (Gomori PAMS) to stain the basement membrane zone (BMZ), and Masson’s trichrome (MT) to stain collagen in the fibrous tissue during the proliferation/remodelation stages of healing [23, 24]. In addition, routine Gram stain was performed to evaluate bacterial proliferation. Systematic microscopic evaluation included re-epithelisation, inflammatory response, BMZ formation, connective tissue formation and remodelling during the healing process.

### Morphometric analysis

For the morphometric analysis, 3 non-sequential sections (HE stained) from each one of the 8 wound biopsies (4 treated and 4 control) taken at 2, 7, 14, 21 and 28 DPW were used. From each section, three 40X high magnification fields (HMF) at the lateral edges and bed wound were photographed; thus, for each control point and group, two pathologists scored the inflammatory cells (heterophils, macrophages, lymphocytes and fibroblasts) in 36 photographs. The morphometric analysis was performed with the software “Image Pro Plus 4.0 software” (Media Cybernetics, Silver Spring, MD, USA).

### Statistical analyses

The distribution of the variable in all data columns was analysed by the Kolmogorov-Smirnov test. Mean wound contraction in both groups, expressed as the percentage of perimeter reduction from the initial wound, was compared at each time point by an unpaired t test. As cells counts were not normally distributed, the non-parametric Kruskal-Wallis test and the Dunn’s post-test for multiple comparisons were used to compare the number of heterophils, macrophages, lymphocytes and fibroblasts at each time point. A value of *P* < 0.05 was considered significant. The Grubbs’ test was used to detect significant outliers. After finishing the experiment, we had a 95% power at day 28 to detect differences between means of 18.28% with a significance level (alpha) of 0.05 (two-tailed). Sample power was determined using GraphPad StatMate 2.00 for Windows. All other statistical calculations and graphics were performed using the Prism 5.04 software for windows (GraphPad Software Inc., San Diego, California).

## Results

### Clinical evaluation of wound healing

**Fig. 1:**
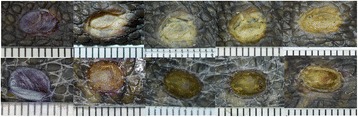
Sequence of clinical photographs illustrating wound contraction and crust macroscopic features in two representative wounds from each group. Wounds were recorded and measured digitally every week from day 0 to 28. Upper row corresponds to control group. Lower row corresponds to insulin-treated group (figures were rotated to align internal scales in millimetres)

All the animals completed the study since the experimental procedure was well tolerated and the induced wounds did not show any remarkable complication. There were no significant differences in gained weight between the insulin-treated and control animals within both groups (data not shown). The biopsy procedure produced well-delimited circular wounds deep enough to allow visualisation of the subcutaneous superficial skeletal muscle (Fig. 1). Immediately after performing the wound, the area was gradually covered with serous or serous-haemorrhagic fluid, but haemorrhages were rare. Insulin application caused the formation of a smooth, waterproof film attached to the edges of the wound.

In Group 1, the wounds were readily covered by a crust that persisted along the 28 days but the macroscopic features of the crusts were clearly different between insulin-treated and controls wounds (Fig. 1). In the control wounds, crusts were characterized by a lighter colour and a more irregular surface. Although the crusts covered all the wound area, most wounds had fissures that exposed the wound bed. Also, the crusts of the control wounds acquired progressively a moistened appearance and at day 28 presented a mucoid texture. By contrast, the insulin-treated wounds were characterized by a darker colour, a more regular surface, and greater consistency manifested by the absence of the crust fissures that were so common in the control wounds. At day 28 the insulin-treated crusts had not developed the mucoid texture observed in the control wounds (Fig. 1).

**Fig. 2:**
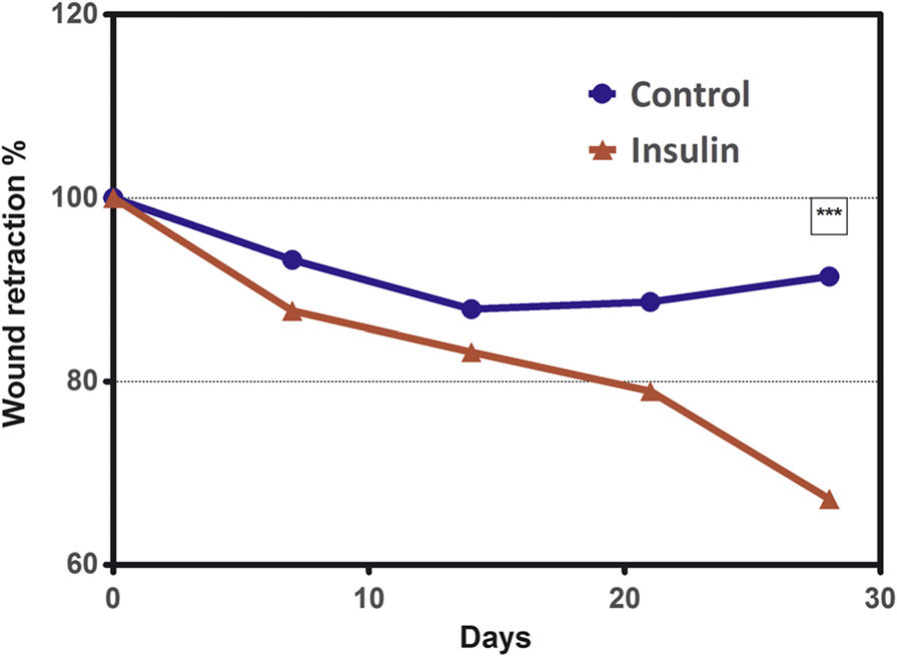
Evolution of wound contraction during the first 28 days post-injury in the control and insulin-treated groups. Each point represents mean wound size expressed as a percentage of the initial wound circumference. Statistically significant differences between groups: *** *P* < 0.001 (unpaired t test)

Mean wound contraction, estimated by the percentage of perimeter reduction from the initial wound was higher in the insulin-treated wounds at every time point (Fig. 2). At days 7 and 14 wound contraction was similar in both groups and also progressed with a similar pace but, after this initial phase, the control wounds reversed this tendency at day 21 and their area slightly increased whereas in the insulin-treated wounds contraction progressed faster, with a more pronounced down slope so that at day 28, when mean wound size in the control and insulin-treated wounds were 91.41% and 67.15% respectively, differences were very significant (*P* < 0.0001) (Table 1).

**Table 1:** Descriptive statistics of wound size at each time point expressed as a percentage of the initial wound circumference

	7 DPW (T1)		14 DPW (T2)		21 DPW (T3)		28 DPW (T4)	
	Control	Insulin	Control	Insulin	Control	Insulin	Control	Insulin
Number of values	24	24	24	24	24	24	24	24
Mean	93.24	87.66	87.84	83.19	88.64	78.93	91.41	67.15
Std. Deviation	11.98	15.76	12.45	15.16	17.75	16.63	19.42	13.89
Std. Error	2.445	3.217	2.542	3.095	3.623	3.395	3.964	2.835
Lower 95% CI of mean	88.18	81.01	82.58	76.79	81.15	71.90	83.21	61.28
Upper 95% CI of mean	98.30	94.32	93.10	89.59	96.13	85.95	99.61	73.01
Minimum	68.76	65.02	59.96	62.42	56.05	57.48	52.56	39.95
25% Percentile	84.09	75.46	78.66	72.32	74.43	67.01	74.37	54.94
Median	96.44	84.13	87.79	80.17	88.27	77.12	99.98	65.48
75% Percentile	101.0	95.64	98.01	89.19	102.1	88.96	104.4	75.60
Maximum	116.4	132.1	113.2	130.5	124.6	119.4	132.1	94.76

Wound size was registered weekly during 4 weeks; DPW: days post-wounding

### Microscopic findings

#### Control skin

Biopsies obtained at wound-induction time were used as internal controls; normal skin structure consisted in a regularly thick epidermis (15 to 25 keratinocytes layers), differentiated into three strata: *stratum germinativum* (basal), *stratum spinosum* or suprabasal and *stratum corneum*. The dermis and hypodermis of the limbs were thin and collagen bundles, disposed in parallel to the epidermis, represented the main component of the extracellular matrix (ECM). Fibrocytes and other resident cells as histiocytes and lymphocytes were scarce or inconspicuous throughout the collagen bands; melanocytes were variable in number, arranged according to the turtles’ pigmentation pattern, and regularly located in the outer dermis around dermal vessels as well as within the basal keratinocytes. Dermo-epidermal junction was defined by a thin and smooth BMZ that was observed as a faint homogeneous or fibrillar bluish band using MT stain and brown-blackish strip using methenamine silver stain.

#### Histological evaluation of wound healing

**Fig. 3:**
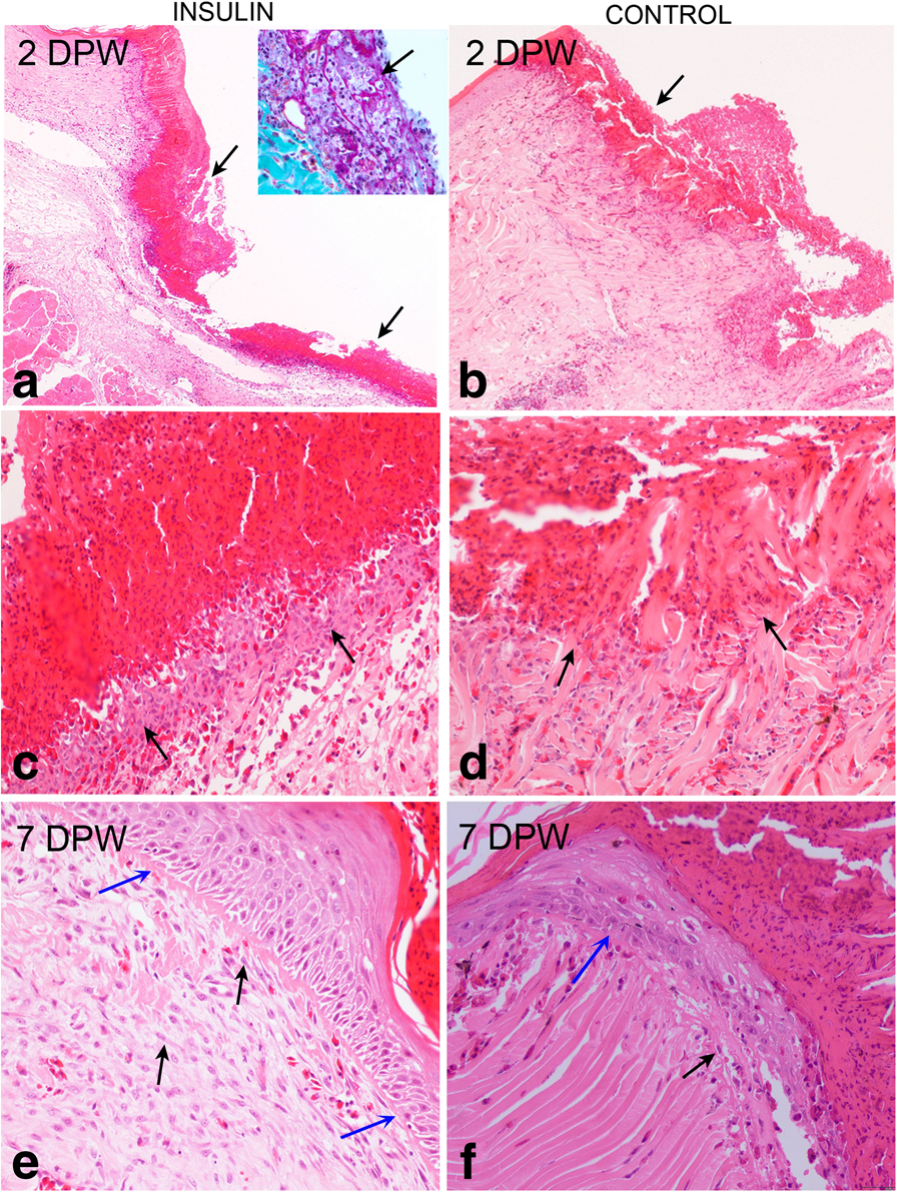
The histology of red-eared slider turtle skin. Sections taken from the lateral and bed margins of the wound in insulin-treated (**a**) and control (**b**) turtles at 2 DPW. There is a noticeable palisade of acidophilic plasma, fibrin (*arrows*) and heterophils exudate. At higher magnification, note that infiltrating macrophages (*arrows*) are more prominent in insulin-treated wound bed edges (**c**) that in control wounds (**d**). At 7 DPW re-epithelisation of the lateral edges is observed in both groups (**e** and **f**), but insulin-treated wounds show a better differentiated epidermal strata and BMZ (**e** and **f**; *blue arrows*) as well as higher number of fibroblasts (**e**, *black arrows*) than control wounds (**f**, *black arrows*) in the granulation tissue

After 2 DPW, insulin-treated and control wounds showed similar microscopic features; the wound surface was covered by an incipient crust composed by plasma and fibrin admixed with numerous heterophils and erythrocytes (Fig. 3a to d). The lateral and deep wound edges of both groups were infiltrated by a mild to moderate exudate of plasma, fibrin (Fig. 3a, inset), intact or degranulated heterophils, and variable quantity of macrophages. Macrophages were more numerous and outlined more clearly the wound margins in treated that in non-treated wounds (Fig. 3c and d). At the perilesional dermis and subcutis the inflammatory reaction was similar in both groups and consisted in moderate hyperaemia, interstitial oedema, perivascular infiltrate of heterophils and scant or moderate number of small lymphocytes.

At 7 DPW, insulin-treated and control wounds appeared covered by a serous-cellular crust formed by a dense acidophilic exudate of plasma, fibrin, numerous heterophils, erythrocytes and cell debris. Re-epithelisation was quite similar in both groups, only the upper half of the lateral edges was covered with a new stratified epithelium under the crust (Fig. 3e and f), but differentiation of the epidermal strata was better in insulin-treated wounds (Fig. 3e). Concurrently with new epidermis formation, the BMZ was more defined in the treated wounds (Fig. 3e). Besides, in both groups, the lateral margins and wound bed were occupied by a variable quantity of inflammatory cells and granulation tissue (new small blood vessels and proliferation of fibroblasts) that was most abundant in the treated wounds and scarce in some control wounds (Fig. 3e and f).

**Fig. 4:**
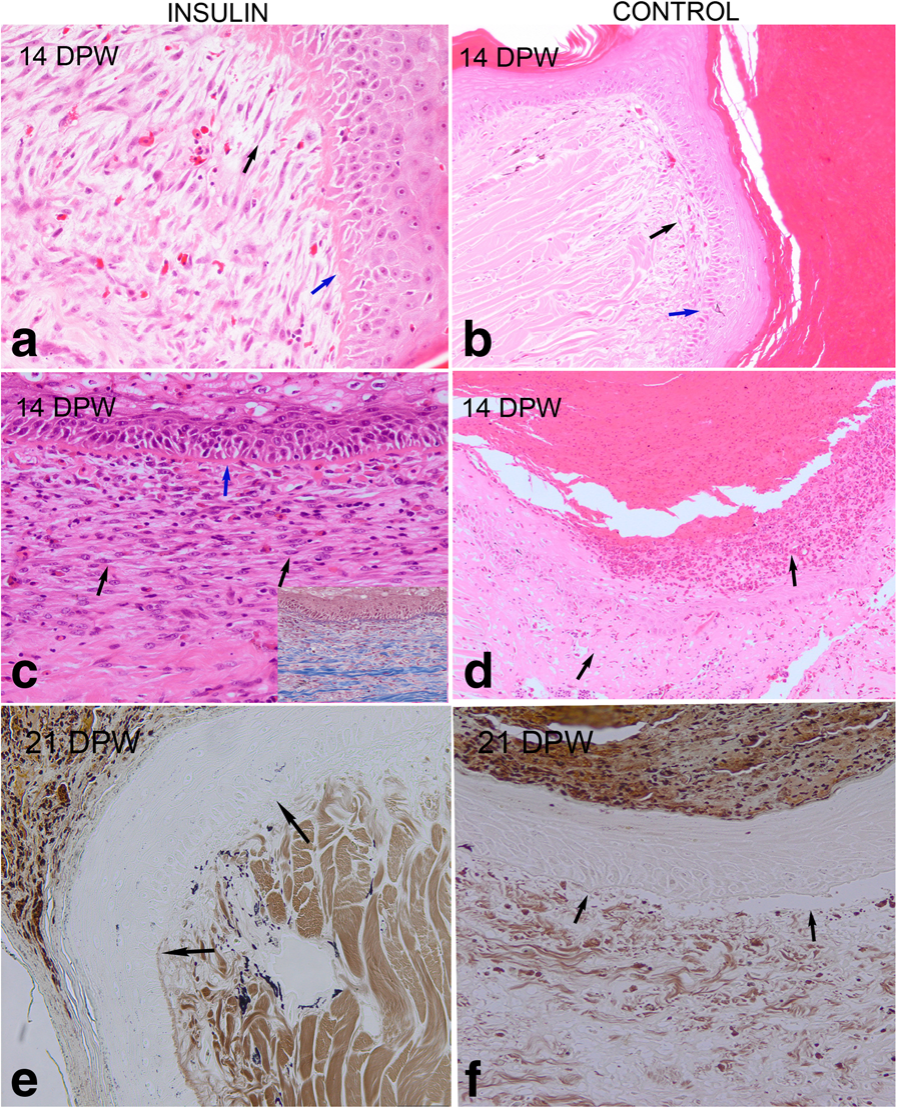
The histology of red-eared slider turtle skin. Sections taken from the lateral and bed margins of the wound in insulin-treated and control turtles at 14 DPW. Insulin-treated wounds show abundant granulation tissue characterized by large fibroblasts (*black arrows*) disposed perpendicular (**a**, lateral margin) or parallel to the epidermis (**c**, wound bed); the BZM is well defined (**a** and **c**, *blue arrows*). Haematoxylin-eosin. Moderate quantity of collagen bundles (*blue stained*) are observed in the regenerate dermis (**c**, inset). Masson’s trichrome. At this time point, the granulation tissue (**b**, *black arrow*) and the BMZ (**b**, *blue arrow*) are less developed in control wounds and the inflammatory reaction more active (**d**, wound bed), characterized by oedema, heterophils and macrophages infiltration (*arrows*). Haematoxylin-eosin stain. At 21 DPW, the BMZ is better differentiated in insulin-treated wounds (**e**) than in control wounds (**f**) in which the epidermis was frequently detached (*arrows*)

At 14 DPW, a dense crust covered both treated and control wounds; re-epithelisation was complete in the majority of wounds but in the insulin-treated group the dermo-epidermal union was firmer and better defined by the BMZ (Fig. 4a and c). Treated wounds showed an abundant granulation tissue under the new epidermis with large fibroblasts, frequently disposed in parallel to the surface, and a better structured BMZ than in the control counterparts (Fig. 4a to d); consequently, detachments of the new epidermis were observed in the control wounds but were rare in the insulin-treated wounds. The inflammatory infiltrate was abundant in treated wounds, both in the lateral edges and in the wound bed and was characterized by a moderate quantity of plasma and fibrin, fewer heterophils and macrophages and less prominent lymphocytes. The control group showed a similar cellular infiltrate but with less heterophils, macrophages and fibroblasts (Fig. 4b and d).

At 21 DPW, all wounds were still covered by an acidophilic crust with abundant cell debris. The new epidermis was thicker and well differentiated, and showed some degree of hyperplasia in both insulin-treated and control wounds (Fig. 4e and f). The BMZ was more complete and the granulation tissue was most mature in the insulin-treated wounds. At this time point, detachment of the epidermis was observed in the control wounds (Fig. 4f). The inflammatory infiltrate decreased in treated wounds, but lymphocytes and fibroblasts were increased or similar in the control group.

**Fig. 5:**
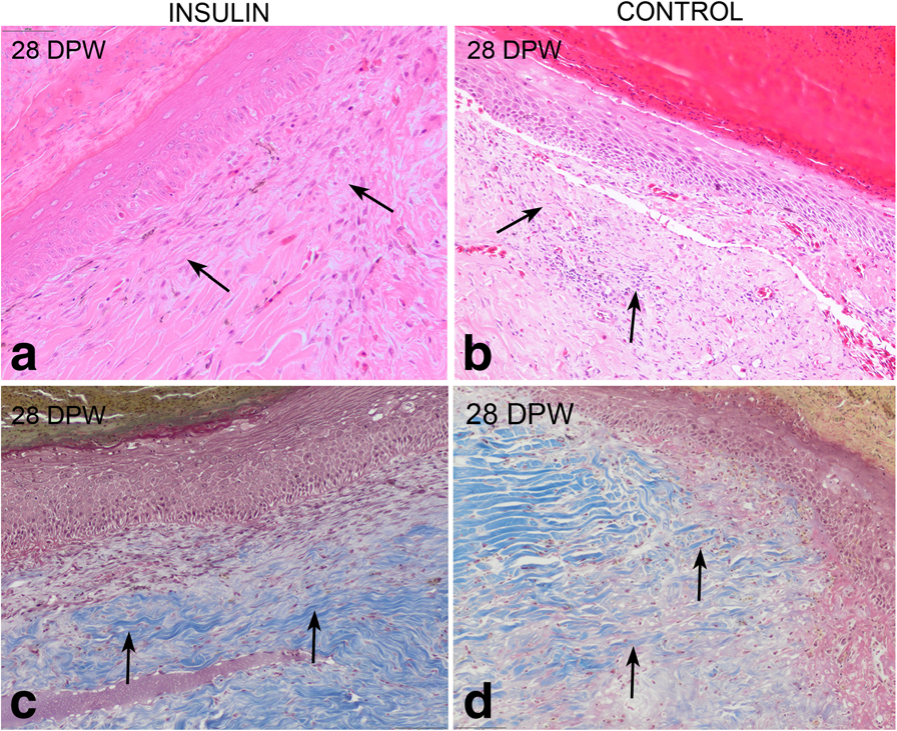
The histology of red-eared slider turtle skin. Sections taken from the bed margins of the wound in insulin-treated and control turtles at 28 DPW. Insulin-treated wounds show abundant collagen deposition and remodelation (**a** and **c**, *arrows*); control wounds show persistent inflammatory exudate and immature granulation tissue with scarce collagen deposition (**b** and **d**, *arrows*), Haematoxylin-eosin (**a** and **b**) and Masson’s trichrome (**b** and **d**) stains

At 28 DPW, insulin-treated wound edges were occupied by mature granulation tissue characterized by a lower number of large fibroblasts and more abundant and thicker collagen bands (Fig. 5a and c), that were arranged in a pattern resembling the normal dermal connective tissue (disposed parallel to surface). In the control wounds, the granulation tissue was more cellular and immature, with more fibroblasts and a variable quantity of collagen fibres (Fig. 5b and d). The BMZ was well developed across the dermo-epidermal union but in the bed zone the methenamine silver was paler than in the margins and normal skin; this finding suggests that the BMZ was still immature both in treated and control wounds at this time point. Repigmentation was moderated and progressed from the lateral edges. From 7 to 28 DPW the granulation tissue consistently had fewer vascular small blood vessels (angiogenesis) both in treated and control wounds. Occasional Gram positive coccaceous bacterial colonies were observed in the surface of the scabs but did not reach the healing tissue.

#### Morphometric analysis

**Fig. 6:**
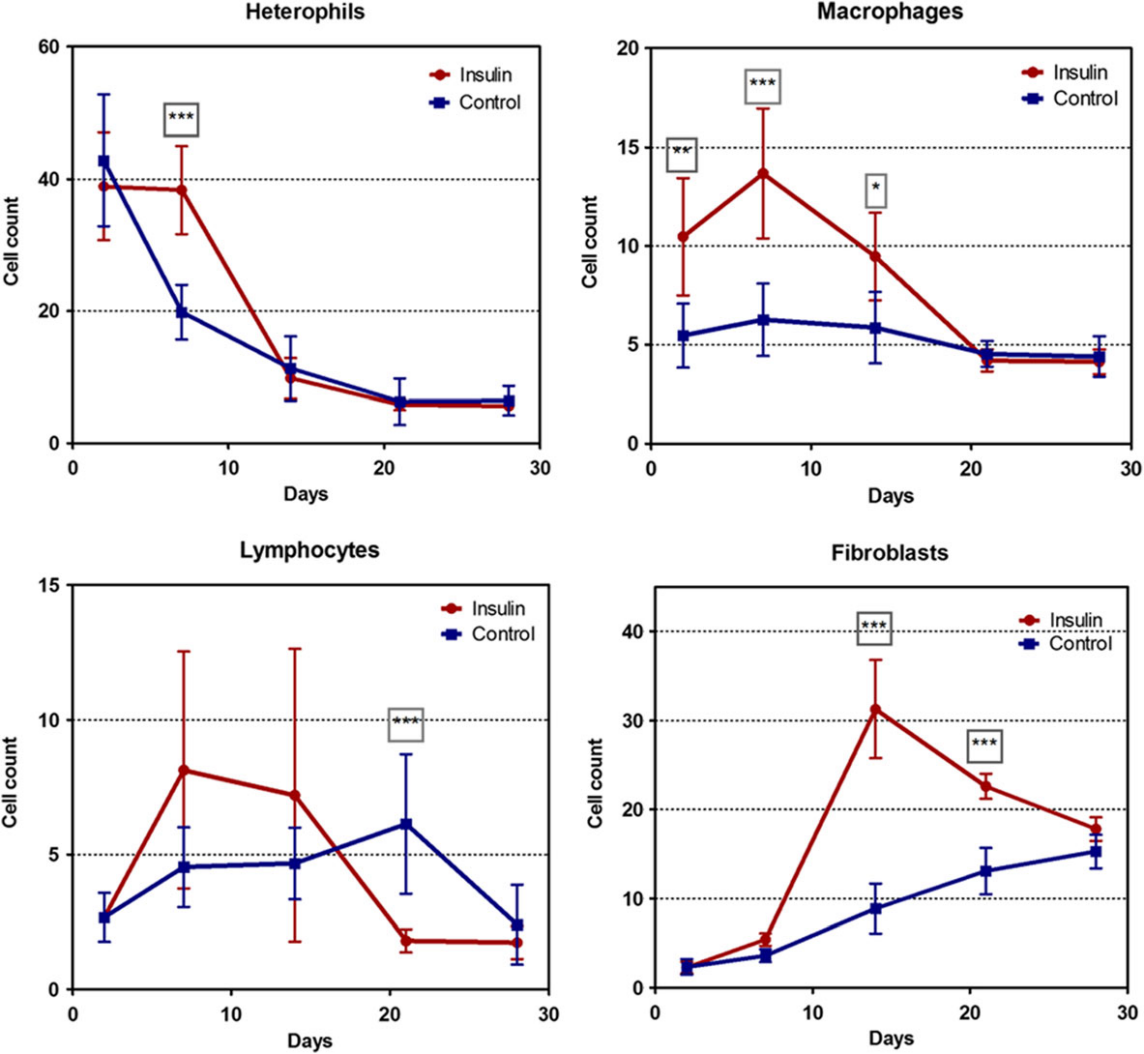
Mean counts of inflammatory cells at each time point from 2 to 28 days after wound creation. Vertical bars represent standard deviation. Statistically significant differences between groups: * *P* < 0.05; ** *P* < 0.01; *** *P* < 0.001 (Kruskal-Wallis test with Dunn’s Multiple Comparison Test)

The topical application of insulin resulted in statistically significant differences in the mean counts of heterophils, macrophages, fibroblasts and lymphocyte counts at different time points along the 2 to 28 days follow up time (Fig. 6). At day 2 the mean number of heterophils was similar in both groups but the control wounds showed a rapid decline during the following days, whereas in the insulin-treated wounds the number of heterophils remained unchanged through the first week of healing and differences in mean counts were highly significant at day 7 (*P* < 0.0001). After this first week, the density of heterophils evolved similarly in both groups. The effect of insulin was especially accentuated on the macrophage counts. Insulin application was associated with significantly higher macrophage mean counts during the first 2 weeks of wound healing reaching the highest differences at 7 DPW (*P* = 0.0002). Regarding fibroblast counts, insulin administration was associated with significantly higher mean counts at day 7 (*P* = 0.025) but especially at day 14 (*P* < 0.0001) and 21 (*P* < 0.0001). Finally, lymphocyte mean counts followed a different pattern since the non-treated wounds showed significantly higher counts at 21 DPW (*P* < 0.001) because lymphocyte density decreased rapidly after 14 DPW in the insulin treated wounds (Fig. 6).

## Discussion

In a previous study we found that cutaneous second-intention wound healing in healthy turtles (*Trachemys scripta elegans*), exposed to daily variations in ambient temperature and with free access to water, progresses slowly and with an indolent behaviour [10]. Under the same experimental conditions topical insulin significantly modified the inflammatory response and improved second-intention wound healing of skin in turtles. One week administration of daily topical insulin resulted clinically in higher mean wound contraction and microscopically increased number of heterophils (the reptile equivalents of mammalian neutrophils) [14], macrophages and fibroblasts at different times along the cicatrisation process. Also, crust formation and macroscopic aspects of the wound were improved, although this observation has to be considered subjective.

Insulin is a polypeptide highly conserved phylogenetically in all vertebrates including reptiles. Antiserum specific for porcine insulin has been used to identify turtle β cells by immunohistochemistry [12] and to measure insulin concentrations in turtle pancreas extracts by radioimmunoassay [13]. This homology between vertebrate insulin allowed us to choose porcine insulin for the present study. Insulin regulates a variety of biological processes including protein turnover, glucose transport, hemodynamics, and fatty acid metabolism. The result is a beneficial profile of anabolic and anticatabolic effects which are important in second intention healing [15, 25–27]. Cutaneous wound healing is a dynamic and complex physiologic process, as tissue repairing involves pleiotropic molecular and cellular events. The initial clot stops the bleeding and facilitates the migration of inflammatory cells, which are attracted by growth factors, cytokines and chemokines released into the area. Subsequent inflammation is essential in the early phases of wound healing; first neutrophils and later macrophages infiltrate the margins of the incision and release proteolytic enzymes than serve to clean out debris and proliferating bacteria [28]. However, the persistence of inflammation leads to delayed healing in ulterior phases [29]. In the current study, topical insulin induced morphological changes that resulted in statistically significant higher mean numbers of heterophils, macrophages and fibroblasts at several time-points, and was associated with a significantly lower density of lymphocytes at 21 DPW in the inflammatory infiltrate. These findings demonstrate that topical insulin has the capacity to modify the inflammatory response of turtle skin after injury, in accordance with previous studies in non-diabetic mammals [15, 16, 18, 25, 26, 30].

In this experimental study topical insulin significantly increased the number of heterophils until 7 DPW. Few studies have investigated the influence of topical insulin on neutrophils during wound healing. Chen et al. [31] reported suppressed infiltration of neutrophils and decreased healing time in mice wounds treated with topical insulin. These results can be considered contradictory since early infiltration of neutrophils is an essential first step of the healing process and the improved healing was attributed to an insulin-induced increased function of mice neutrophils. In contrast, we found a higher number of heterophils during the first 7 DPW coinciding with insulin application, and this increase in heterophils was associated with higher numbers of macrophages and with faster wound contraction at 28 DPW. Although reptile heterophils are considered equivalent to mammals’ neutrophils [14], our discrepancies with the report of Chen et al. [31] could be explained by a different cellular response of reptile heterophils to topical insulin as well as by differences in study design as in mice, neutrophil counts were determined just during the first 3 days after injury.

Previous reports in mammals have demonstrated that macrophages produce several growth factors and cytokines that stimulate fibroblasts and keratinocytes resulting in enhanced granulation tissue formation and keratinocyte migration (re-epithelisation) [28]. Our results, both for macrophages and fibroblasts, are consistent with these studies. Chen et al. [11] reported that insulin induced a noticeable increased infiltration of macrophages in the first 3 days after wounding, as macrophage number in the insulin-treated wounds was the same at day 2 as at day 3 in the control wounds. A similar response was seen in the present study on turtles. The enhanced macrophage infiltration into the wound area indicates that insulin promotes wound healing by up-regulating wound inflammatory response, specifically the quantity and function of macrophages. The effect of topical insulin would be comparable with cytokines with the ability to activate macrophages, such as interleukin 1β and macrophage-activating lipopeptide-2 that have been successfully used to promote wound closure [32, 33].

The dynamics of lymphocyte mean counts was similar to that of macrophages with higher means at 7 and 14 DPW but differences were not statistically significant due to the high standard deviation of the data. Probably, a larger sample might have achieved statistical significance. Nevertheless, a particular feature of the lymphocytic response was the pronounced drop in lymphocyte counts from day 14 to day 21 DPW that, coinciding with an increase of the mean lymphocyte count in the control group, resulted in a significant lower mean count of lymphocytes in the treated group. In our opinion, the faster and higher increase of lymphocytes and the earlier reduction of the lymphocytic infiltrate in the treated wounds are another indication of the positive effect topical insulin exerted on the inflammatory response.

The significant increase in fibroblast mean counts found in turtle insulin-treated wounds is in agreement with previous findings in mammals [16–18, 26]. Faster wound healing and increased wound tensile strength was observed in rats treated with topical insulin, and histological analysis of wound tissue revealed an earlier appearance of collagen fibres with more compact, dense and well-oriented morphology compared with control animals [15]. In amphibians (*Ambystoma mexicanum*) it was shown that insulin can stimulate sulphate uptake and growth of cartilage as in mammals [34]. The mechanisms by which insulin exerts these effects are not fully understood but it is known that insulin can stimulate a variety of cellular functions important in tissue repair including collagen synthesis in skin fibroblasts [35]. In general, growth factors such as insulin can act as chemoattractants to recruit important cells such as leukocytes and fibroblasts into the wound area, stimulating angiogenesis, ECM formation and degradation, and cytokine release [18, 36, 37]. On the other hand, re-epithelisation was quite similar in both groups, although since 7 DPW insulin-treated wounds showed a better differentiated and defined new stratified epithelium and BMZ respectively. Previous research in mammals has demonstrated that topical insulin improves tissue repair through stimulation of keratinocyte migration and insulin signalling pathways [38, 39]. In their study, Liu et al. [39], observed that the skin wounds of rats treated topically with insulin healed faster, the surface cells in the epidermis covered the wound more quickly, and the cells in the dermis rebuilt blood vessels more rapidly. It was also shown that topical insulin stimulated the proliferation and migration of keratinocytes and the migration of microvascular endothelial cells.

The ability of topical insulin in turtles to increase the number of fibroblasts would be a major contribution to explain the faster wound contraction observed in treated animals. Wound contraction is a major component of second-intention wound healing and the pivotal feature for contraction is granulation tissue formation and remodelling [7, 8, 28]. As has been described in snakes [6], turtles form a persistent dried crust over the wound bed and healing is characterized by epithelialization under the crust which decreases in thickness as the dermis fills the skin defect, meanwhile contraction is very limited [10]. Thus, the significantly higher wound contraction at day 28 in the insulin-treated wounds is a remarkable finding that could be explained by the ability of topical insulin to modulate the inflammatory response increasing macrophage infiltration, granulation tissue and collagen deposition in turtles as has been described in mammals [16, 30]. At day 28 mean wound contraction in the insulin-treated group was 24.26% higher than in the control group. A greater difference after 28 days would have been desirable but in our opinion is large enough to consider topical insulin potentially useful, alone or combined with other growth factors, in the treatment of non-healing wounds of reptiles. One weakness of the present study was its preclinical condition; for practical reasons we could not include diseased animals or animals with non-healing wounds. However, as the potential benefit of insulin or other growth factors has not been investigated before in skin wounds of reptiles, a preclinical study was necessary before undertaking clinical studies to determine the real usefulness of topical insulin.

Depending on its concentration, the amount of topical insulin may have systemic effects. Previous studies have revealed the benefits of topical insulin treatment for injuries to healthy and diabetic mammals [11, 16, 18, 26], but the concentrations used have been variable. In rats, a recent study assayed different concentrations of insulin and determined that the doses that induced the best effect in wound healing were 0.5 IU and 1.0 IU/100 g. The dose of 1.0 IU/100 g, in some animals, induced alterations in plasma glucose. Therefore, a cream with a concentration of 0.5 IU/100 g was used for all experiments [16]. However, another study also in rats used topically regular Humulin® (Eli Lilly Turkey, Istanbul, Turkey) at a concentration of 100 IU/mL without any systemic effect [18]. The use of vehicles that enhance insulin absorption and provide longer contact times may explain the striking differences in insulin doses between studies. In the present study, we chose glycerol as vehicle because it is soluble with aqueous insulin, hygroscopic, cosmetically acceptable and has a high viscosity index. In preliminary experiments, we tested different concentrations of insulin and found that, compared with non-treated animals, a concentration of 5 IU/ml administered for 1 week improved wound contraction without influencing plasma glucose concentrations. As a topical solution of this concentration provided a sufficient local effect, higher concentrations were not assayed. A daily application during the first week of cicatrization was considered practical and appropriate since the inflammatory reaction occurs rapidly after the injury and because turtle skin wound healing is characterized by epithelialization under a heavy crust that prevents a longer insulin application.

Second intention cutaneous healing in mammals is characterized by fibrosis, and lack of hair follicles and glands in the regenerated dermis [9]. The ability to heal without scarification has been studied in several species of reptiles with different outcomes. Recently, in leopard geckos, 3 mm full thickness skin wounds to the tail and body resulted in a scar free healing characterized by a delay in blood vessels formation and no fibrosis; the authors concluded that a proportional vascular response was a key factor to avoid scar formation [40]. In the present report, 6 mm full thickness skin wounds healed by granulation tissue formation resulting in fibrosis still present after 28 days, both in insulin treated and control wounds. This finding was in agreement with our previous study describing normal skin wound healing in the same turtle species. Although the epidermis and pigmentation of the healed area was indistinguishable from non-damaged skin, variable degrees of fibrosis were still present in the restored dermis after 135 days post-wounding [10]. In other reptiles such as common garter snakes, skin wounds also form scar tissue and scale regeneration is incomplete [6], as has been reported in a more recent research on the regeneration of scales in tail and body skin of lizards [41]. In this study, using a similar full thickness model of skin wounds, either in lizards able to tail regeneration (*A. carolinensis*) or in lizards poorly capable of tail regeneration (*I. iguana*), the new scales were irregular and smaller than normal and showed a different shape and pigmentation compared with normal scales [41].

## Conclusions

We found that topical insulin application improved second-intention wound healing in skin of turtles exposed to daily variations of ambient temperature and in aquatic environment. The results indicated that insulin promotes healing by regulating wound inflammatory response, specifically the number and activity of macrophages, heterophils and fibroblasts at early stages of wound healing (0 to 21 DPW). Therefore, topical insulin is a potentially useful treatment for poorly healing skin wounds of turtles and should be evaluated in non-experimental wounds of turtles and other reptiles.

## Abbreviations

ATR: Appropriate temperature range
BMZ: Basement membrane zone
DPW: Days post-wound
ECM: Extracellular matrix
HMF: High magnification fields
